# Association between homocysteine, C-reactive protein, lipid level, and sleep quality in perimenopausal and postmenopausal women

**DOI:** 10.1097/MD.0000000000028408

**Published:** 2021-12-23

**Authors:** Hongyan Zhang, Qianwen Wang, Miao Deng, Yijie Chen, Wenhua Liu, Jian Huang, Zhifen Zhang

**Affiliations:** a Department of Obstetrics and Gynecology, Nanjing Medical University, Nanjing, Jiangsu, China; b Department of Reproductive Medicine Center, Hangzhou Women's Hospital (Hangzhou Maternity and Child Health Care Hospital), Hangzhou, Zhejiang, China; c The Fourth Clinical School of Zhejiang Chinese Medicine University, Hangzhou, Zhejiang, China.

**Keywords:** C-reactive protein, homocysteine, lipid level, menopause, sleep quality

## Abstract

This study aimed to investigate the correlation between homocysteine (HCY), C-reactive protein (CRP), lipid levels, and sleep quality in perimenopausal and postmenopausal women.

We collected data from 217 patients (perimenopause and postmenopausal) who visited the gynecological endocrine outpatient department of our hospital between January 2017 and January 2019. The quality and patterns of sleep were measured using the Pittsburgh Sleep Quality Index, and relationships between HCY, CRP, lipid levels, and sleep quality were analyzed according to a Pittsburgh Sleep Quality Index ≥ 8.

There were significant differences in age, education level, and occupation among patients with different levels of sleep quality (*P* < .05). HCY, CRP, total cholesterol, triglyceride, and low-density lipoprotein levels were significantly higher in patients with poor sleep quality than in those with good sleep quality (*P* < .05). Age, education level, occupation, HCY, CRP, and lipid levels (total cholesterol, triglyceride, low-density lipoprotein, high-density lipoprotein) were all significant influencing factors for sleep quality in perimenopausal and postmenopausal women (all *P* < .05). After adjusting for age, education level, occupation, HCY, and CRP levels were all significant and independent risk factors for sleep quality in perimenopausal and postmenopausal women (all *P* < .05).

Levels of HCY, CRP, and lipids were significantly correlated with sleep quality in perimenopausal and postmenopausal women. HCY and CRP were identified as independent risk factors for sleep quality in perimenopausal and postmenopausal women, thus providing theoretical support for the clinical improvement of sleep quality.

## 1 Introduction

Worldwide, it is estimated that there are approximately 470 million women over the age of 50 and that approximately 25 million women enter the menopausal transition (MT) period each year.^[1]^ The MT period is considered a high-risk period for female sleep disorders. Approximately 40% to 60% of women suffer from sleep disorders or insomnia; this is one of the most common health problems affecting perimenopausal and postmenopausal women and can seriously affect their quality of life.^[2]^

Homocysteine (HCY) is a sulfur-containing amino acid cysteine that is synthesized by the liver and plays a role in the metabolism of methionine. Recent studies have demonstrated that HCY is closely associated with many diseases. Serum HCY levels are higher in postmenopausal women than in younger women.^[3]^ Increased levels of HCY are unfavorably associated with coagulation function and the vasodilatory and antithrombotic action of nitric oxide, thereby increasing the risk of thrombosis and acute myocardial infarction.^[4]^

C-reactive protein (CRP) is a sensitive marker for acute inflammatory response and an independent risk factor for cardiovascular disease. Postmenopausal women are also known to have higher CRP levels than younger women because of the lack of cardioprotective effects for estrogen.^[1,5]^

Previous studies have found that sleep disorders are associated with obesity, cardiovascular disease, diabetes, and mood disorders during MT.^[6,7]^ However, little is known about the specific correlation between sleep disorders and HCY, CRP, and lipid levels during MT. In this study, we aimed to investigate the relationships between HCY, CRP, lipid levels, and sleep quality, in perimenopausal and postmenopausal women to provide clinical intervention and health guidance strategies for improving sleep quality and reducing the risk of metabolic syndrome and cardiovascular disease.

## 2 Methods and measurements

### 2.1 Data source and collection

A total of 657 peri- and postmenopausal women were evaluated in the gynecological endocrine outpatient department of Hangzhou Women's Hospital between January 2017 and January 2019. Of these women, 217 were enrolled in this study. To be considered for inclusion in this study, participants needed to be aged 40 to 60 years and experienced menstrual changes or perimenopausal symptoms (based on the Kupperman Menopausal Index) or had gone through menopause (early postmenopause: first 1–5 years after final menses, late menopause >5 years after the final menstrual period). Patients who had undergone hysterectomy were excluded, as were those who had previously received menopausal hormone therapy.

We also excluded women who were pregnant or lactating, those receiving antidepressants, hypnotics, phytoestrogens, and menopausal hormone therapy, and those with a mental illness, abnormal breast mass, and/or any abnormality in the thyroid.

This study was approved by the Ethics Committee of Hangzhou Women's Hospital (Hangzhou Maternity and Child Health Care Hospital). Women were free to participate in the study and were enrolled after providing written and informed consent. All of the collected information was kept confidential.

### 2.2 Measurements

Participants were investigated using the Pittsburgh Sleep Quality Index (PSQI)^[8,9]^; which is a self-administered instrument that measures subjective sleep quality during the preceding month. In brief, 18 items were included in the PSQI that were used to weigh scores for the following 7 components: subjective sleep quality, sleep latency, sleep duration, habitual sleep efficiency, sleep disturbance, use of sleeping medications, and daytime dysfunction. Each component was judged on a scale of 0 to 3: none, <1 time/week, 1 to 2 times/week, or ≥3 times/week, respectively. The scores for the 7 components were summed to generate a total global PSQI score (range, 0–21). A PSQI score of 8 or higher was defined as sleep disorder. Patients were then divided into 2 groups: PSQI score < 8 (n = 112) and PSQI score ≥ 8 (n = 105).

### 2.3 Blood sample analysis

Fasting venous blood samples were collected from all subjects and centrifuged at 3500 rpm for 10 minutes at room temperature. Samples were stored at –80°C until analysis. Blood cell analysis was performed using an automatic blood analyzer (UniCelDxH 800, BECKMAN). Serum levels of HCY, CRP, and lipid indices were measured using commercially available kits on an autoanalyzer by enzyme chemiluminescent immunoassay (Siemens Healthcare Diagnostics, ADVIA1800, Siemens AG, Germany).

### 2.4 Statistical analysis

All statistical analyses were performed using SPSS version 19.0 (IBM Corporation, Armonk, NY). All variables were tested for normality using the Kolmogorov-Smirnov test. We also used Levene test to analyze the homogeneity of variance. Data are presented as mean ± standard deviation or as numbers (%). Continuous variables were compared using the independent *t* test (if data from the 2 groups satisfied the test for homogeneity of variance); otherwise, data were tested using the Mann-Whitney test. Categorical variables were compared using the Wilcoxon rank-sum test. The associations between HCY, CRP, and sleep disorders (PSQI ≥ 8) were determined by logistic regression analysis. Statistical significance was set at *P* < .05. The logistic regression model was assessed using the Hosmer-Lemeshow test.

## 3 Results

The mean age of the patients showing good sleep quality (PSQI < 8) was 47.02 ± 5.04 years. The mean age of the patients with poor sleep quality (PSQI > 8) was 49.87 ± 5.48 years. There were significant differences between the 2 groups in terms of age, education level, and occupation (*P* < .05). Marital status and menopausal status were not significantly different between the 2 groups (*P* > .05). The sociodemographic characteristics of the participants are presented in Table 1.

**Table 1:** Factors associated with 2 sleep quality groups.

	PSQI < 8 (n = 112)	PSQI > 8 (n = 105)	*X* ^2^ */t*	*P* value
Age	47.02 ± 5.04	49.87 ± 5.48	3.99	<.001
40–45	41.12 ± 3.63 (33)	42.69 ± 2.60 (26)	1.86	.068
46–50	47.78 ± 1.37 (54)	48.30 ± 1.49 (30)	1.62	.108
51–55	52.27 ± 1.20 (22)	53.06 ± 1.48 (33)	2.17	.03
56–60	59.67 ± 0.58 (3)	57.75 ± 1.61 (16)	1.99	.06
Education level				
Junior high school or below	29 (26)	41 (39)	7.757	.021
Secondary school	43 (38)	43 (41)		
College diploma or above	40 (36)	21 (20)		
Occupation				
Work	87 (78)	63 (60)	10.995	.004
Departure	8 (7)	23 (22)		
Retirement	17 (15)	19 (18)		
Marital status				
Married	110 (98)	101 (96)	0.244	.621
Divorced/widowed/single	2 (2)	4 (4)		
Menopausal status				
Perimenopause	55 (49)	39 (37)	3.485	.175
Early postmenopause	51 (46)	61 (61)		
Late postmenopause	6 (5)	5 (5)		

PSQI = Pittsburgh Sleep Quality Index.

Table 2 shows that HCY, CRP, triglyceride (TG), total cholesterol (TC), low-density lipoprotein (LDL), and fasting insulin (FINS) levels were significantly higher in the group with poor sleep quality than the group with good sleep quality (*P* < .05). High-density lipoprotein (HDL) was significantly lower in the group with poor sleep quality (*P* < .05), while body mass index, waist/hip ratio, fasting blood glucose level, systolic blood pressure, and diastolic blood pressure, were not significantly different when compared between the 2 groups (*P* > .05).

**Table 2:** HCY, CRP and lipid levels in 2 sleep quality groups.

	PSQI < 8 (n = 112)	PSQI > 8 (n = 105)	*t*	*P* value
BMI	21.89 ± 2.63	22.46 ± 2.21	1.72	.087
WHR	0.84 ± 0.06	0.85 ± 0.06	1.25	.213
TC	4.86 ± 0.91	5.39 ± 0.98	4.13	<.001
TG	1.08 ± 0.74	1.36 ± 0.58	3.09	<.001
HDL	1.76 ± 0.40	1.44 ± 0.29	6.52	.001
LDL	2.68 ± 0.63	3.15 ± 0.90	4.42	<.001
FBG	4.95 ± 0.48	5.00 ± 0.45	0.74	.463
FINS	6.43 ± 5.26	10.02 ± 8.26	3.80	<.001
HCY	9.70 ± 2.42	11.49 ± 2.84	5.03	<.001
CRP	2.31 ± 1.61	3.41 ± 2.08	4.34	<.001
SBP	117.72 ± 11.74	116.99 ± 9.55	0.50	.616
DBP	73.59 ± 7.58	74.05 ± 7.11	0.46	.647

BMI = body mass index, CRP = C-reactive protein, DBP = diastolic blood pressure, FBG = fasting blood glucose, FINS = fasting insulin, HCY = homocysteine, HDL = high-density lipoprotein, LDL = low-density lipoprotein, PSQI = Pittsburgh Sleep Quality Index, SBP = systolic blood pressure, TC = total cholesterol, TG = triglyceride, WHR = waist/hip ratio.

As shown in Table 3, univariate logistic regression suggested that age, HCY, CRP, TG, TC, HDL, and LDL were strongly associated with poor sleep quality (*P* < .05). We further investigated the independent risk factors for sleep disorders using binary logistic regression and found that age (odds ratio [OR]: 1.095; 95% confidence interval [CI]: 1.015–1.182), HCY level (OR: 1.412; 95% CI: 1.202–1.659), CRP (OR: 1.266: 95% CI: 1.020–1.571), TC (OR: 2.542; 95% CI: 1.231–5.248), and HDL (OR: 0.033; 95% CI: 0.009–0.127) were related to sleep quality and were identified as independent risk factors for poor sleep quality (*P* < .05).

**Table 3:** Influencing factors of sleep quality in (peri-)postmenopausal women.

	Univariate analysis		Multivariate analysis	
Factors	*OR (95% CI)*	*P* value	*OR (95% CI)*	*P* value
Age	1.111 (1.051–1.174)	.000	1.095 (1.015–1.182)	.019
HCY	1.357 (1.185–1.553)	.000	1.412 (1.202–1.659)	.000
CRP	1.398 (1.185–1.648)	.000	1.266 (1.020–1.571)	.032
TG	2.107 (1.289–3.445)	.003	0.974 (0.506–1.874)	.936
TC	1.802 (1.337–2.427)	.000	2.542 (1.231–5.248)	.012
HDL	0.069 (0.026–0.179)	.000	0.033 (0.009–0.127)	.000
LDL	2.155 (1.492–3.114)	.000	1.003 (0.442–2.272)	.995
FINS	1.085 (1.036–1.135)	.000	1.153 (1.079–1.233)	.000

CI = confidence interval, CRP = C-reactive protein, FINS = fasting insulin, HCY = homocysteine, HDL = High-density lipoprotein, LDL = low-density lipoprotein, OR = odds ratio, TC = total cholesterol, TG = triglyceride.

## 4 Discussion

Epidemiological studies have shown that a significant number of middle-aged women experience a gradual deterioration of sleep quality during the MT,^[10]^ and such sleep problems have been associated with a poor quality of life. Sleep disturbance is one of the most common symptoms in 50% to 80% of perimenopausal women. In our analysis of women experiencing MT with different sleep qualities, we identified a significant correlation between age structure and sleep disorders and that those aged 51 to 55 years were the most significantly affected. In addition, we identified that age was an independent risk factor for sleep disorders during the MT period, which may be related to the fluctuations in endogenous hormone levels and vasomotor symptoms in women.

Previous studies have demonstrated that HCY is associated with menopause status. Serum HCY levels gradually increase across the MT period to the postmenopausal period because of the extreme fluctuations in estrogen and FSH levels during the MT stage.^[11]^ HCY has been confirmed to be a strong inflammation-inducing factor that can cause endothelial cell damage and is an independent risk factor for cardiovascular disease.^[12,13]^ Increased levels of HCY predispose to endothelial injury, stimulate HDL oxidation through an increase in the activity of methionine synthetase, and affect the proliferation of endothelial smooth muscle cells. Previous studies have also indicated that the levels of HCY in postmenopausal women are higher than those in young women.^[14]^ Sanchez-Espinosa et al^[15]^ were the first to demonstrate that sleep mediated the association between HCY and oxidative status in mild cognitive impairment. However, few studies have investigated the association between the combination of HCY and sleep disorders and MT.

In the present study, we investigated the association between HCY and sleep quality among a large sample of middle-aged women transitioning through menopause. According to the sleep quality groups, the serum levels of HCY were significantly higher in the group experiencing poor sleep quality than those in the group experiencing good sleep quality. Univariate regression analysis showed that increased levels of HCY significantly interfered with sleep disorders during the menopause transition, and that indicators of poor sleep quality were independently associated with HCY after adjusting for demographic factors such as age.

Sleep represents a biologically plausible, modifiable, and underexplored pathway through which the MT may increase the risk of metabolic syndrome and its consequential influence on cardiometabolic disease. Hall et al^[16]^ suggested that the dimensions of sleep that change during MT are linked with metabolic syndrome. For instance, women who sleep for less than 6 hours are more likely to develop metabolic syndrome. Chen^[17]^ showed that sleep disturbances, including obstructive sleep apnea (OSA), sleep deprivation, and sleep fragmentation, are involved in the development of metabolic syndrome. Our results are consistent with these earlier findings.

Our study also revealed that metabolism-related indices, such as LDL, TC, and TG levels, were significantly higher in menopausal women with poor sleep quality, thus implying that sleep quality was significantly correlated with metabolism. After adjusting for age and other factors, regression analysis showed that HDL and TC were independent risk factors for sleep quality.

Accumulating evidence indicates that CRP, an essential indicator of the inflammatory process, has been identified as an independent risk factor for cardiovascular and cerebrovascular diseases.^[5]^ However, it is unclear whether the coexistence of elevated CRP and HCY levels accelerates the risk of sleep disorders during the MT period. The body is in a state of chronic low-grade inflammation after menopause, which is caused by abnormalities in LDL and oxidative stress indices (HCY, CRP) that can further affect sleep quality. Ozen et al found that obese patients with OSA syndrome may have an increased rate of metabolic syndrome and higher levels of serum lipids, fasting glucose, FINS, leptin, fibrinogen, and CRP than obese subjects without sleep apnea. Meng et al^[5]^ performed a meta-analysis and reported that patients with OSA had higher CRP levels than the control group, and the CRP levels of patients with a higher body mass index and apnea-hypopnea indices were significantly different, thus supporting the fact that CRP is a useful biomarker for OSA. Irwin et al^[18]^ performed a cohort study and meta-analysis and showed that sleep disorders were associated with increased levels of 2 systemic inflammatory markers, interleukin-6 and CRP; women with sleep disorders had significantly higher levels of CRP than men. The Study of Women's Health Across the Nation sleep study^[19]^ found a correlation between sleep quality and inflammatory factors (such as CRP and fibrinogen) in pro-coagulation pathways, which mediated the relationships between sleep characteristics and cardiometabolic health. A shorter sleep duration was associated with higher CRP and fibrinogen levels. Nowakowski et al^[20]^ investigated the relationship between sleep continuity, sleep duration, and inflammatory biomarkers in middle-aged women during menopause transition. The results indicated that the quality and duration of sleep were independently correlated with the inflammatory biomarkers. However, Taheri et al^[21]^ performed a large cohort study and found no significant correlation between subjective/objective factors of sleep duration and CRP. The relationship between sleep quality and inflammatory markers in menopausal women remains controversial. Logistic regression analysis, carried out in the present study, showed that age, education level, HCY, CRP, lipid levels, and FINS levels were the main factors affecting the sleep quality of menopausal transitional women, which is in accordance with previous studies.^[15,17]^ After adjusting for the effects of age, education level, and menopausal status, HCY and CRP levels were identified as independent risk factors affecting sleep quality.

This study showed that serum levels of HCY, lipids, and CRP were higher in the sleep disorder group than in the normal group. Correlation analysis further showed that CRP and HCY, robust biomarkers of underlying systemic inflammation, were independently associated with indicators of poorer sleep quality after controlling for confounding factors such as age and demographic factors. However, the exact progressive cause remains unknown with regard to the correlation between HCY, CRP levels, and sleep disorders during the MT period. Further randomized studies are needed to confirm these associations. It is also important to identify the precise mechanisms involved.

## Acknowledgments

The authors thank all team members for their contributions to the study.

## Author contributions

JH and ZZ designed the study. QW, MD, and HZ acquired the data. YC and WL analyzed the data.

QW and HZ were responsible for this methodology. HZ and ZZ drafted the manuscript. JH and ZZ reviewed and edited the manuscript.

**Data curation:** Qianwen Wang.

**Methodology:** Qianwen Wang, Miao Deng, Yijie Chen, Wenhua Liu.

**Project administration:** Jian Huang.

**Resources:** Wenhua Liu, Zhifen Zhang.

**Writing – original draft:** Hongyan Zhang.

**Writing – review & editing:** Jian Huang, Zhifen Zhang.
